# Influenza B virus infection alters the regenerative potential of murine alveolar type 2 pneumocytes

**DOI:** 10.1128/mbio.02743-24

**Published:** 2024-12-31

**Authors:** Satoko Nakano, Cait E. Hamele, Aleksandra Tata, Purushothama Rao Tata, Nicholas S. Heaton

**Affiliations:** 1 Department of Molecular Genetics and Microbiology, Duke University School of Medicine, Durham, North Carolina, USA; 2 Department of Cell Biology, Duke University School of Medicine, Durham, North Carolina, USA; 3 Department of Surgery, Duke University School of Medicine, Durham, North Carolina, USA; 4 Division of Pulmonary, Allergy, and Critical Care Medicine, Department of Medicine, Duke University School of Medicine, Durham, North Carolina, USA; 5 Duke Regeneration Center, Duke University School of Medicine, Durham, North Carolina, USA; 6 Duke Human Vaccine Institute, Duke University School of Medicine, Durham, North Carolina, USA; 7 Duke Center for Virology, Duke University School of Medicine, Durham, North Carolina, USA; McMaster University, Hamilton, Ontario, Canada; Johns Hopkins University Bloomberg School of Public Health, Baltimore, Maryland, USA

**Keywords:** epithelial cell infection, lung epithelial repair, survival of direct infection, regeneration

## Abstract

Respiratory epithelial cells can survive direct infection by influenza viruses, and the long-term consequences of that infection have been characterized in a subset of proximal airway cell types. The impact on the cells that survive viral infection in the distal lung epithelia, however, is much less well-characterized. Utilizing a Cre-expressing influenza B virus (IBV) and a lox-stop-lox tdTomato reporter mouse model, we identified that alveolar type 2 (AT2) pneumocytes, a progenitor cell type in the distal lung, can survive viral infection. We show that survival of infection is associated with transcriptional dysregulation compared to bystander AT2 pneumocytes from the same lung. Furthermore, *ex vivo* experiments revealed a significant reduction in proliferation rates in survivor AT2 pneumocytes compared to matched, non-directly infected bystander cells. Our findings not only enhance our understanding of the AT2 pneumocyte response to IBV infection but could also have broader implications for the mechanisms of respiratory epithelial repair post-viral infection.

**IMPORTANCE:**

Alveolar type 2 (AT2) pneumocytes are a cell type critical for repair of the distal lung after an injury, such as a viral infection. After epithelial damage, AT2 pneumocytes proliferate for both self-renewal and differentiation into type I pneumocytes to repopulate the epithelium. Theoretically, some of the long-term lung sequelae associated with viral infections could be the result of inappropriate AT2 behavior. Here, the authors report that during an influenza B virus infection, some of the actively infected AT2 pneumocytes can ultimately eliminate all traces of the viral RNA and persist in the host long term. As a consequence of having been infected, however, the cells display an altered transcriptional profile and decreased proliferative capacity. These data together suggest a mechanism for how an acute viral infection can have long-term impacts on the pulmonary system.

## INTRODUCTION

Influenza causes significant morbidity and mortality each year, with an estimated 290,000 to 650,000 people succumbing to influenza-associated respiratory disease annually (1). Upon infection with the influenza virus, individuals experience acute respiratory symptoms. Once the virus is cleared, the respiratory epithelia undergo repair and return to normal healthy conditions. However, this is not always the case, and in some situations, if the infected individual is unable to properly repair the epithelia, they experience post-viral sequelae (2). The idea of post-viral sequelae has been recently gaining attention due to the effects reported by the coronavirus disease 2019 pandemic, but this concept in influenza viral infection has been reported as early as after the 1918 influenza pandemic (3). Understanding potential viral manipulations of respiratory epithelial cells as it affects lung repair after influenza infection would be highly impactful for the future development of therapeutics to mitigate post-viral sequelae.

Historically, it was widely believed that during an acute viral infection, all cells that were infected with influenza virus would die either intrinsically or due to the actions of the immune system, and, thus, only uninfected bystander cells would remain. Data from numerous groups, however, now show that many cell types that were directly infected can persist well after viral clearance (4–6). We have subsequently investigated the impact of surviving influenza virus infection on various airway epithelial cell types and have demonstrated roles for survivor cells in the innate immune response and epithelial regeneration after infection (7–9). While these studies show the effects that infected cells can have post-viral clearance, they represent only a partial understanding of the function of survivor respiratory epithelial cells, as numerous other epithelial cell types that survive influenza virus infection, especially those in the distal alveoli, have yet to be characterized.

Despite the virus primarily interacting with proximal airway cells during the initiation of infection, severe pulmonary distress is often correlated with viral spread to the distal lung epithelia (10, 11). The distal lung consists of two distinct alveolar pneumocytes, AT1 and AT2, which play pivotal roles in respiratory function. AT1 pneumocytes regulate gas exchange, and AT2 pneumocytes maintain surfactant levels and act as progenitor cells during alveolar injury (12, 13). When the alveoli experiences damage, normally quiescent AT2 pneumocytes begin to proliferate for self-renewal or differentiate into AT1 pneumocytes to replenish lost cells (14). This process is critical in maintaining the epithelial barrier after influenza virus infection, which helps to mitigate secondary bacterial infections that can be major drivers of morbidity and mortality (15, 16). AT2 pneumocyte dysregulation and loss have been reported in several respiratory diseases, such as idiopathic pulmonary fibrosis (17), chronic obstructive pulmonary disease (18–20), and acute respiratory distress syndrome (21, 22). Interestingly, while dysregulation and loss of AT2 pneumocytes in respiratory disease have been well-documented in the literature, the underlying causes often remain elusive. Thus, the cellular survival of viral infection could represent a previously unreported mechanism contributing to AT2 pneumocyte dysregulation.

In this study, we found that both of the distal respiratory epithelial cell types, AT1 and AT2 pneumocytes, can survive influenza infection at some frequency. Further characterization of the surviving AT2 pneumocytes revealed that they were transcriptionally distinct from their bystander counterparts. Specifically, surviving AT2 pneumocytes downregulate pathways that regulate development, proliferation, and differentiation, which we could measure phenotypically in an *ex vivo* model where we observed survivor AT2 proliferation to be reduced compared to bystander cells. While there are some anatomical differences between the respiratory systems of different mammalian species, the basic cellular structures of the distal lung parenchyma are largely consistent, generally enabling findings and advances from murine alveolus studies to be applied to humans (23). Based on these data, we propose a model in which virally induced proliferation dysregulation in surviving AT2 pneumocytes could be a meaningful contributor to the repair rates of the lung epithelia post-viral clearance.

## RESULTS

**Fig 1:**
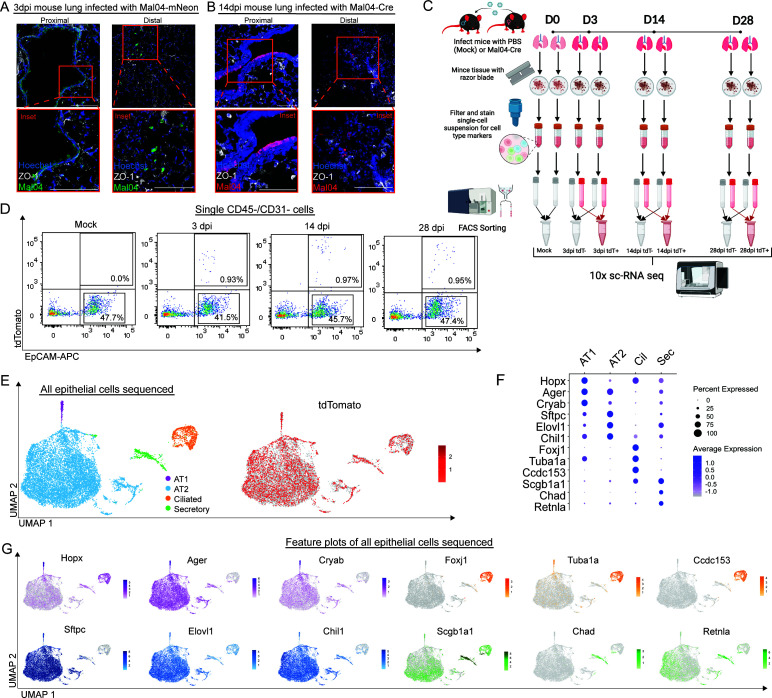
Distal airway epithelial cells can survive infection by Mal04 and be identified using scRNA-seq. **A**) Confocal microscopy of the 3dpi lung section infected with Mal04-mNeon (green) and stained with Hoescht 33342 (blue) and ZO-1 (white) to define cell boundaries. **B**) Confocal microscopy of 14 dpi lung section infected with Mal04-Cre (red) and stained with Hoescht 33342 (blue) and ZO-1 (white). **C**) Diagram of 10× sc-RNA seq experiment cell collection and processing (*n* = 8, 2 mice were pooled for each time point). D) Representative flow cytometry of survivor cells collected at the indicated time points following Mal04-Cre infection in Cre-inducible tdTomato reporter mice. **E**) UMAP dimensionality reduction plot showing all epithelial cells sequenced. Clusters were identified using specific cell type markers. **F**) Dot plot displaying the expression of specific cell type markers used to determine cell type clusters in **FIG. 1E**. **G**) Feature plots of genes displayed in **FIG. 1F**. Clusters are colored based on their cell type determined by the expression of specific cell type markers. All scale bars = 100 µm.

It is known that influenza A viruses have a broad cellular tropism within the respiratory epithelia and infect several cell types, including basal cells, proximal ciliated and club cells, as well as more distal AT1 and AT2 pneumocytes (24). Similarly, influenza B viruses (IBVs) have also been reported to infect a wide range of cell types within the respiratory epithelia (25); however, the cellular tropisms are generally less well characterized. To better understand the regions of the lung that would be affected by a contemporary, Victoria-lineage IBV in mice, we infected animals with a B/Malaysia/2506/2004 (Mal04) strain that harbors the fluorescent reporter protein, mNeon-Green (9, 26). Lungs were collected and sectioned at 3 days post-infection (dpi) to understand rates of infection in the proximal versus the distal lung (Fig. 1A). While we observed different amounts of infected cells in the proximal lung compared to the distal lung epithelia, there were obvious regions of infected cells across all major regions of the lung. To understand the ultimate fate of these infected cells, lox-stop-lox-tdTomato reporter mice from The Jackson Laboratory were infected with a sub-lethal dose of a Mal04 variant that expresses Cre-recombinase during infection (9, 26). This method would allow for any cell that experiences infection to be permanently labeled with tdTomato. Lungs were collected and sectioned at 14 dpi (a timepoint post-viral clearance) (9) to compare the number of survivor cells in the proximal and distal lungs compared to the number of cells initially infected (Fig. 1B). As expected, we find regions of surviving cells generally matched regions observed with active infection, and populations of surviving cells were apparent in the distal lung.

Our previous work and the lung sections indicated that we would observe cell type heterogeneity across the surviving cell populations; we, therefore, designed a single-cell RNA sequencing (scRNA-seq) experiment (Fig. 1C) to understand what cell types survive infection by IBV in more detail. With a particular emphasis on collecting the cells of the distal lung, we used fluorescence-activated cell sorting to isolate bystander and survivor epithelial cells at Days 0 (Mock), 3, 14, and 28 post-infection (Fig. 1D) and performed scRNA-seq. Out of all the collected lung cells, about 1% of the total lung cells expressed tdTomato (Fig. 1D). As a first analysis step, we aggregated all our samples and performed unbiased clustering, which we rationalized would allow for the segregation of major cell types (Fig. 1E). Indeed, the major clusters were segregated by key cell type markers (Fig. 1F and G), indicating that our protocol captured the expected cell types. While we attempted to collect primarily distal lung cells, we observed that AT2 pneumocytes dominated the population likely due to the physical characteristics of AT1 pneumocytes decreasing the efficiency of their capture. Outside of identifying major cell types, we also used tdTomato as a marker to distinguish the bystander cells from infected and survivor cells. In all cell type clusters, including distal AT1 and AT2 pneumocytes, we observed cells that are positive for tdTomato, suggesting that all cell types can be infected and survive infection (Fig. 1E). Due to our technical ability to capture a large AT2 population and the importance of AT2 pneumocytes during and after viral infection, we decided to focus further efforts on understanding the effects of surviving viral infection in that population.

**Fig 2:**
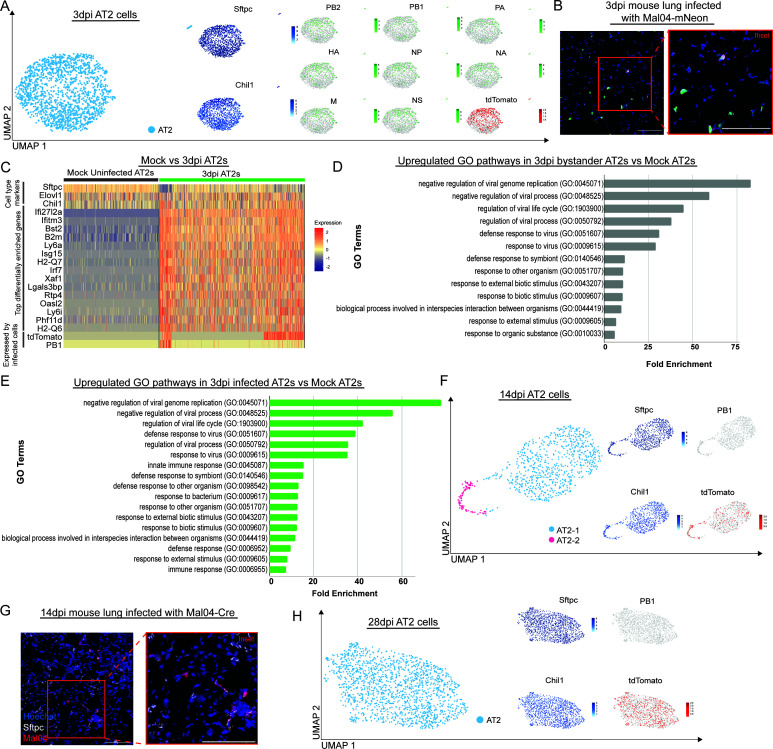
AT2 pneumocytes are permissible and can survive infection by Mal04. **A**) UMAP dimensionality reduction plot showing all 3 dpi AT2 cells sequenced. To the right are feature plots of the eight viral segments of Mal04-Cre and tdTomato. **B**) Confocal microscopy of 3 dpi lung section infected with Mal04-mNeon (green) and stained with Hoescht 33342 (blue) and Sftpc (pink). **C**) Heatmap displaying AT2 cell type marker expression, with the top genes differentially enriched in 3 dpi AT2 pneumocytes compared to mock uninfected AT2 pneumocytes (excluding viral segments, avg_log_2_FoldChange < −3, p_val_adj < 1.0e−150) and genes expected to be expressed by infected pneumocytes (tdTomato and PB1). **D**) Top upregulated gene ontology (GO) terms were plotted between mock uninfected and 3 dpi bystander AT2 pneumocytes (fold enrichment > 5 and false discovery rate (FDR) *P*-value < 2.0E−04). **E**) Top upregulated GO terms were plotted between mock uninfected and 3 dpi infected AT2 pneumocytes (fold enrichment > 5, and FDR *P*-value < 2.0E−04). **F**) UMAP dimensionality reduction plot showing all 14 dpi AT2 pneumocytes sequenced. **G**) Confocal microscopy of 14 dpi lung section infected with Mal04-Cre (red) and stained with Hoescht 33342 (blue) and Sftpc (white). **H**) UMAP dimensionality reduction plot showing all 28dpi AT2 pneumocytes sequenced. All scale bars = 100 µm.

We first decided to interrogate the AT2 pneumocyte response to active viral infection. We, therefore, took our 3dpi samples and filtered for AT2 pneumocytes based on cell type-specific markers. We found that both infected and bystander AT2 pneumocytes clustered together largely as one cluster (Fig. 2A, left). The presence of transcripts corresponding to viral genes and/or tdTomato revealed that about half of the collected AT2 cells at this time point were actively or previously infected (Fig. 2A, right). Although we could clearly identify infected AT2 pneumocytes in our scRNA-seq data set, we wanted to confirm this observation via orthogonal assay prior to additional study. Mice were, therefore, infected with Mal04-mNeon, and lungs were collected, sectioned, and stained for the AT2 cell type marker, Sftpc at 3 dpi. As expected, we could identify infected AT2 pneumocytes *in vivo* (Fig. 2B).

Next, to understand how AT2 pneumocytes were responding during infection, we visualized differentially enriched genes between mock uninfected AT2 pneumocytes and all of the AT2 pneumocytes at 3 dpi via heatmap (Fig. 2C). While there is a mixed population of infected, bystander, and survivor AT2 pneumocytes at 3 dpi (identified via the presence or absence of viral RNA and/or tdTomato), most of the top differentially enriched genes in 3 dpi AT2 pneumocytes are known interferon stimulated genes, such as Ifitm3, Bst2, Isg15, and Irf7 (27) (Fig. 2C), consistent with a generally inflammatory state in the lung. Additionally, we could observe a general downregulation of cell type markers in infected cells, consistent with previous reports (28, 29). We also performed gene ontology (GO) analysis on the differentially enriched genes in both bystander and directly infected AT2 pneumocytes compared to mock uninfected AT2 pneumocytes, which revealed that both groups enrich pathways associated with the immune response to infection (Fig. 2D and E).

Next, we wanted to understand the range of AT2 pneumocyte phenotypes post-viral clearance. At 14 dpi, AT2 pneumocytes mostly clustered together; however, a second minor population was also apparent (Fig. 2F). All cells were expressing AT2 cell type markers, Sftpc and Chil1. As expected, we failed to observe the expression of viral RNA at this time point. Evaluation of the tdTomato positive cells across these clusters revealed surviving cells in both clusters, with apparent enrichment in the minor population (Fig. 2F). Again, to confirm the presence of survivor AT2 pneumocytes via an orthogonal assay, we infected Cre-inducible tdTomato reporter mice with Mal04-Cre and collected and sectioned lungs 14 dpi. Consistent with the scRNA-seq data, we found that some, but not all tdTomato positive cells overlapped with AT2 cell marker Sftpc (Fig. 2G). Finally, we wanted to understand the fate of surviving AT2 pneumocytes at a time point post-recovery from viral infection. AT2 pneumocytes are non-terminally differentiated (13, 14) and thus, could theoretically persist long-term in the lung. Indeed, at 28 dpi, we could observe surviving AT2 pneumocytes. However, the overall clustering of the survivor and bystander cells into one group suggested that the heterogeneity observed in the population at 14 dpi had been lost (Fig. 2H).

**Fig 3:**
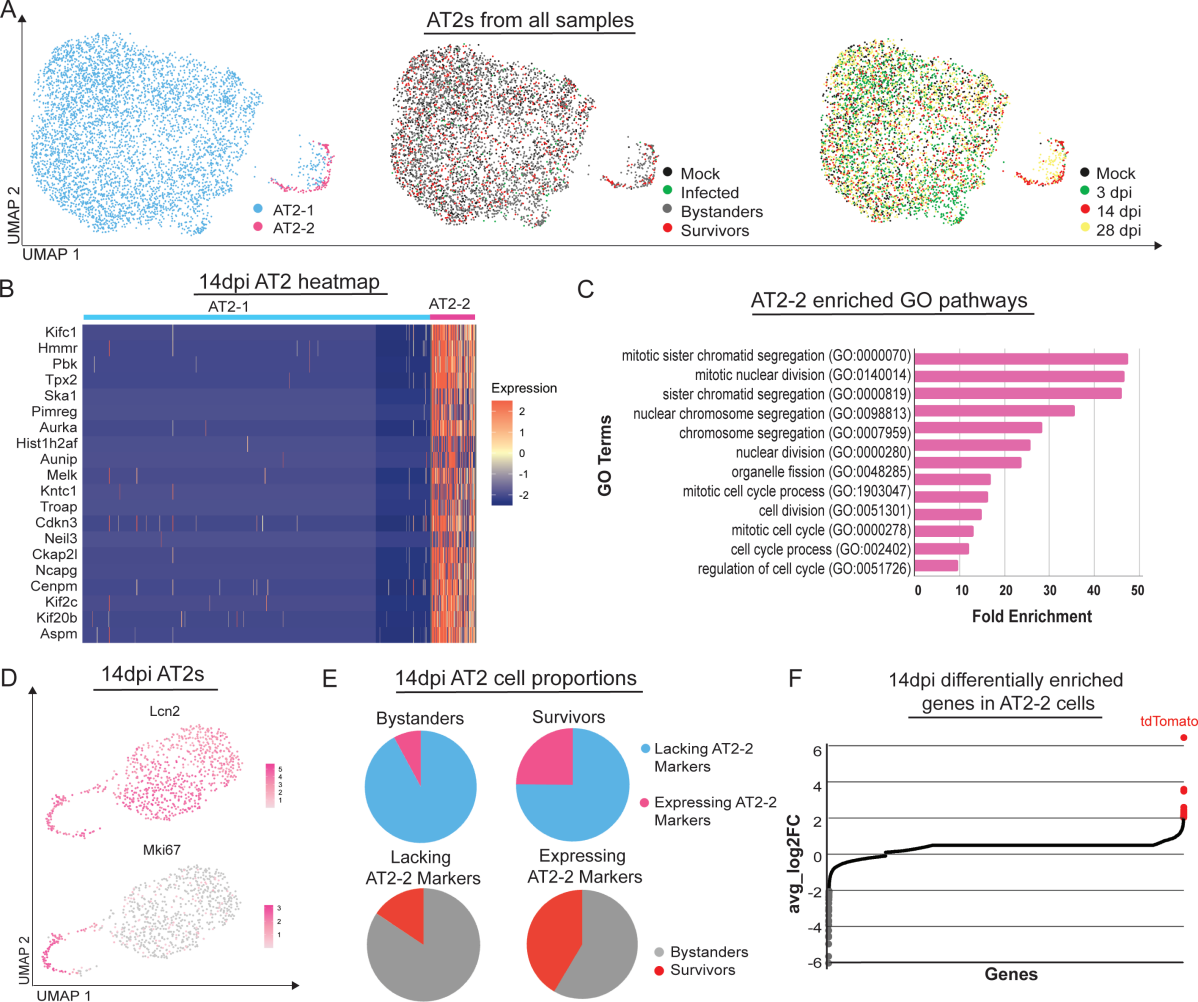
Bystander and survivor AT2 pneumocytes proliferate at 14 dpi. **A**) UMAP dimensionality reduction plot showing all AT2 pneumocytes sequenced from all time points. **B**) Heatmap of the top marker genes (avg_log_2_FoldChange < −5, p_val_adj < 1.0e−100) of AT2-2 identified by FindMarkers function. **C**) Top GO terms (fold enrichment > 5 and false discovery rate *P*-value < 2.5E−05) were plotted using the top marker genes identified in **FIG. 3b**. **D**) UMAP dimensionality reduction feature plots showing all 14 dpi AT2 pneumocytes sequenced displaying expression of genes Lcn2 and Mki67. **E**) Pie graphs displaying cell proportions of bystanders and survivor pneumocytes that are quiescent or cycling and proportion of quiescent and cycling AT2s that are bystander and survivor AT2 pneumocytes. **F**) Plot of all the genes expressed by survivor AT2 pneumocytes compared to bystander AT2 pneumocytes. Genes enriched (avg_log_2_FoldChange > 2) in survivor AT2 pneumocytes are highlighted in red, and those that are enriched (avg_log_2_FoldChange < −2) in bystander AT2 pneumocytes are highlighted in gray.

To better understand the relationship between the AT2 pneumocytes across the infection and recovery time points, we aggregated the AT2 pneumocytes from all samples. One major cluster and one minor cluster were observed (Fig. 3A, left); however, these two clusters did not appear to be driven by infection status (Fig. 3A, middle). When labeling the clusters by time point of collection, however, it became clear that the minor AT2-2 pneumocyte cluster was the same minor cluster observed in the 14 dpi sample earlier (Fig. 3A, right). Although we observed a small number of mock, 3 dpi-, and 28 dpi-associated cells in the AT2-2 cluster, we did not observe separate clusters of cells when analyzed individually by dpi (Fig. 2A and H). We, therefore, restricted subsequent analyses to the 14 dpi AT2 pneumocytes (Fig. 2F) and performed marker analysis on the clusters (Fig. 3B). When we performed the GO analysis on the differentially enriched genes, we found that they were genes related to the cell cycle and proliferation (Fig. 3C). We then looked at the feature plots of Lcn2, a known marker for injury-activated AT2 pneumocytes (30, 31). Interestingly, we saw that nearly all of the AT2 pneumocytes at 14 dpi are expressing some level of Lcn2. However, when looking at the well-established marker for proliferation, Mki67 (32, 33), the proliferation marker is only present in the AT2-2 cluster (Fig. 3D), suggesting that while possibly primed and activated, not all AT2 pneumocytes are actively expressing proliferation markers. To understand the composition of these clusters, we graphed the relative composition of the populations. While survivor cells represent a small proportion of total AT2 pneumocytes collected, survivor AT2 pneumocytes make up almost half of the proliferation markers expressing the AT2 population (Fig. 3E). Finally, because we previously had observed a virally induced dysregulation of gene expression in surviving cells (7, 8), we wanted to determine if the apparently proliferating bystander and survivor pneumocytes were transcriptionally similar. Although the scRNA-seq experiment was not appropriately powered for this type of analysis, we could observe preliminary indications of a small proportion of transcripts that appeared differentially expressed between the two populations (Fig. 3F).

**Fig 4:**
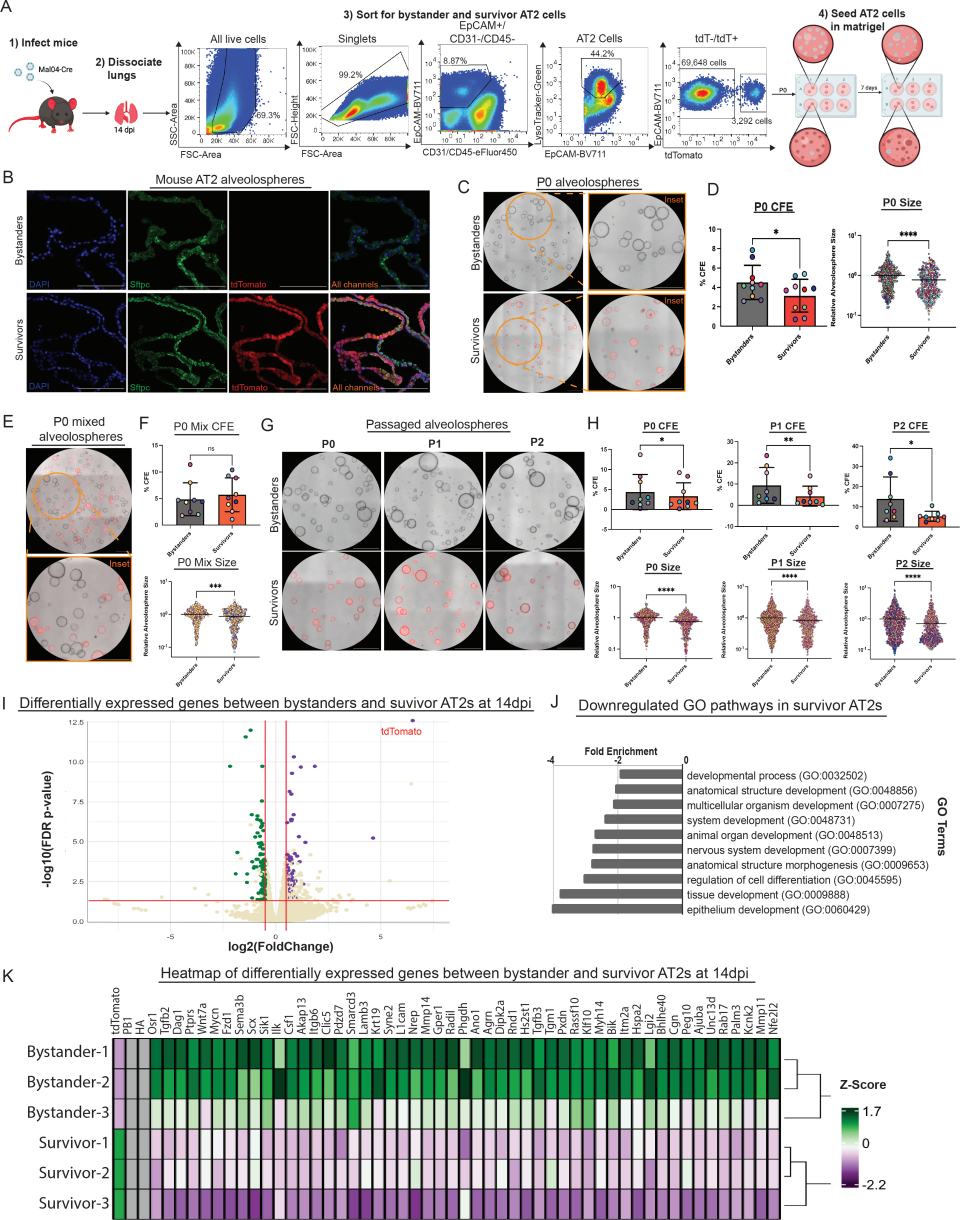
Survivor AT2 pneumocytes proliferate at a slower rate compared to bystander AT2 pneumocytes. **A**) Schematic of the generation of bystander and survivor alveolospheres, including a representative flow cytometry of sorting for bystander and survivor AT2 pneumocytes. **B**) Confocal microscopy of bystander and survivor alveolosphere sections stained with 4′,6-diamidino-2-phenylindole (blue) and Sftpc (green). Survivor pneumocytes are expressing tdTomato (red). Scale bar = 100 µm. **C**) Representative image of bystander and survivor alveolospheres at P0. Scale bar = 1,000 µm. **D**) Quantification of bystander and survivor AT2 colony-forming efficiency (CFE) and alveolosphere sizes at P0 from 10 independent animals. E) Representative image of bystander and survivor alveolospheres when co-cultured for 7 days. Scale bar = 1,000 µm. **F**) Quantification of bystander and survivor AT2 CFE and alveolosphere sizes when co-cultured from nine independent animals. **G**) Representative images of bystander and survivor alveolospheres being passaged from P0 to P1 to P2. Scale bar = 1,000 µm. **H**) Quantification of bystander and survivor AT2 CFE and alveolosphere sizes across P0, P1, and P2 from nine independent animals. **I**) Volcano plot displaying all differentially expressed genes between bystander and survivor AT2 pneumocytes. The horizontal red line represents the false discovery rate (FDR) *P*-value of 0.05, while the vertical red line represents the log_2_fold change of ±0.5. Genes that fell below either of those lines or had a max group mean of less than one are colored in off-white. Genes that are significantly upregulated in survivor cells are labeled in purple, and those that are significantly upregulated in bystanders are labeled in green. **J**) Gene ontology (GO) of the genes upregulated in bystander AT2 cells (log_2_fold change > 0.5, FDR *P*-value < 0.05, max group mean > 1) was conducted, and the top 10 GO terms were plotted (FDR *P*-value < 3.00E−03, and expected genes > 5). **K**) Heatmap of differentially expressed genes between matched bystander and survivor pneumocytes from three animals in developmental and morphogenesis pathways. For panels, including statistics from measuring the CFE, significance was determined using Wilcoxon rank-sum tests. For alveolosphere size, significance was determined using Mann–Whitney tests.

All of the accumulated data thus far suggested that bystander and survivor AT2 pneumocytes can induce a proliferation gene expression profile after clearance of virus; however, there may be transcriptional differences between the two groups. To understand if any virally induced gene expression changes could affect the proliferation behavior, we decided to collect the two populations and culture them *ex vivo* (34). We, therefore, infected Cre-inducible tdTomato reporter mice with Mal04-Cre, collected their lungs at 14 dpi, and sorted bystander and survivor AT2 pneumocytes. Cells were then embedded into Matrigel, and AT2 alveolospheres were cultured (Fig. 4A). To confirm that the alveolospheres were indeed composed of AT2 pneumocytes, we sectioned the alveolospheres and stained for the AT2 cell type marker, Sftpc. We found that nearly all cells are expressed the AT2 marker Sftpc, and the survivor alveolospheres uniquely expressed tdTomato (Fig. 4B). Returning to our original goal of understanding any phenotypic proliferation differences between survivor and bystander AT2 pneumocytes, alveolospheres were grown and quantified for colony-forming efficiency (CFE) and size 7 days after seeding. We found that survivor AT2 pneumocytes have a lower CFE and formed smaller alveolospheres compared to bystander AT2 pneumocytes collected from the same lung (Fig. 4C and D). We also co-cultured bystander and survivor AT2 pneumocytes in the same Matrigel to understand if surviving cells were secreting factors that affected the proliferation rate. Interestingly, while the differences in the CFE were no longer significantly different, the survivor AT2 pneumocytes still formed smaller alveolospheres, suggesting that multiple mechanisms likely contribute to the overall phenotype (Fig. 4E and F). Finally, to determine if the reduced proliferation rate phenotype was transient, we passaged the AT2 alveolospheres twice (Fig. 4G). Across alveolospheres derived from cells collected from different animals, we observed a similar trend as we saw at P0 (Fig. 4H). While the bystander AT2 pneumocytes displayed increased CFE over the passages (consistent with previous reports [35, 36]), survivor AT2 pneumocyte CFE failed to increase with passaging.

Finally, because our earlier transcriptional analysis had insufficient resolution, we collected cultured survivor and bystander AT2 pneumocytes and performed bulk RNA-sequencing to better characterize the differentially expressed genes. A volcano plot representation of the data was consistent with our previous observations that a relatively limited set of genes had significantly altered expression either up or down (Fig. 4I). The GO analysis revealed, however, that the top downregulated genes in survivor AT2 pneumocytes clustered into pathways related to tissue development and cell differentiation (Fig. 4J). Expression levels of the genes involved in these developmental and differentiation pathways were then visualized by a heatmap (Fig. 4K). As expected, only survivor AT2 pneumocytes expressed tdTomato, and neither bystanders nor survivors expressed viral RNA. Many genes related to the Wnt signaling pathway, a key signaling pathway for maintaining stemness in AT2 pneumocytes (37–39), were downregulated, such as *Osr1* (40), *Wnt7a* (37), and *Fzd1* (41). Additionally, there were many other genes downregulated in survivor AT2 pneumocytes that are known to play a role in wound repair in the lungs, such as *Dag1* (42), *Tgfb2* (43), and *Mycn* (44).

## DISCUSSION

While it is appreciated that some respiratory epithelial cells can survive direct infection by viruses, the ability to survive and the full range of effects induced by infection on different cell types are still incompletely understood. In this study, we have confirmed that AT2 pneumocytes can survive infection by Mal04. Furthermore, our data show that while AT2 pneumocytes in general proliferate after viral clearance to repair the damaged epithelia, survivor AT2 pneumocytes fail to proliferate to the same extent as matched bystander counterparts. This dysregulation was maintained over at least two serial passages *ex vivo*, and bulk RNA-sequencing confirmed that survivor AT2 pneumocytes downregulate key pathways known to be critical in AT2 pneumocyte regeneration. Interestingly, the bulk RNA-sequencing data revealed that several genes related to the Wnt signaling pathway were downregulated. Other groups have previously observed that during alveolar injury, AT2 pneumocytes, along with neighboring fibroblasts, activate the Wnt signaling pathway to maintain stemness and facilitate proliferation and transdifferentiation (37). Thus, our survivor AT2 pneumocytes may have dysregulated proliferation due to inappropriate Wnt signaling pathway activation; however, we did not formally test this hypothesis. Irrespective of how viral infection induces AT2 dysfunction, we propose that viral infection can induce long-term effects on the lung epithelia by inducing a “poor-progenitor” phenotype in surviving AT2 pneumocytes.

One interesting question raised by our data is the mechanism of long-term gene dysregulation in surviving AT2 pneumocytes. The inhibited proliferation phenotype was stable over passaging, suggesting an epigenetic basis. We hypothesize that this phenomenon may be similar to the concept of “trained immunity.” This mechanism of immunological memory is induced by epigenetic alterations in the innate immune system, where an initial exposure to a pathogen or inflammatory cytokines “trains” innate immune cells so that their response to subsequent pathogens differs from that of their naïve counterparts (45, 46). This phenomenon has not only been observed predominantly in macrophages and NK cells (45, 46) but has also been reported in respiratory epithelial cells (47) and other cell types (48). Additionally, viral infection has been known to cause epigenetic changes in infected cells, and because cells were thought to be eventually killed by infection, their effects on the immediate immune response has been the main focus of understanding the effects of virally induced epigenetic alterations (49–51). In survivor cells, these changes could affect their canonical behavior and potentially affect lung repair and regeneration long term.

While this study has identified a novel role of survivor cells in respiratory epithelial repair, many important outstanding questions remain. First, the mechanisms of the long-term phenotypic alterations of surviving AT2 populations remain completely unknown and in the future will need to be investigated. Additionally, we have exclusively used the mouse model of influenza virus infection, and it remains unclear if human AT2 pneumocytes will be affected in identical ways. Furthermore, we have used only one strain of virus in this study; hence, future studies to understand the full range of viruses that can induce similar phenotypes will be important in understanding the breadth of impact these types of cell alterations can have. Perhaps, most importantly, however, future studies need to be done to understand the effects of the dysregulation of AT2 pneumocyte proliferation *in vivo*. Survivor cells are relatively rare in the lung, and their contribution to tissue or organ level phenotypes, such as respiratory function, remains unclear. Answers to these questions will allow for a better understanding of how impactful virally induced cellular dysfunction contributes to disease either in the acute pneumonia or recovery phase.

In summary, while it was once generally believed that respiratory epithelial cells were killed after acute viral infection, we now know that there are clear populations of cells that are intrinsically able to clear the virus and persist in the host long term. These cells have the potential to provide a mechanistic link between an acute infection and long-term effects on health. However, future work is needed before such relationships can be established. In the future, a full understanding of the consequences of the survival of infected cells on overall health and recovery after influenza virus infection may provide an avenue for the development of a novel class of therapeutics designed to to enhance alveolar repair after influenza infection and mitigate long-term sequelae.

## MATERIALS AND METHODS

### Mouse lines

B6.Cg-Gt(ROSA)26Sortm14(CAGtdTomato)Hze/J (tdTomato) mice were originally purchased from The Jackson Laboratory and propagated in house.

### Viruses

Influenza B virus (B/Malaysia/2506/2004) with one strain expressing Cre recombinase and another expressing mNeon was generated and utilized, as previously published (9, 26). The viral segments HA, NA, and PB1 were amplified, and 5′ and 3′ ends were sequenced to confirm the correct sequence for each segment in each virus.

### Mouse infections

For all infections, mice were 8–12 weeks old and anesthetized with 100 µL of ketamine–xylazine and intranasally infected with 40 µL of virus at a sub-lethal dose of 75,000 PFU diluted in pharmaceutical-grade phosphate-buffered saline (PBS). Body weight was measured each day over the course of infection with a humane endpoint reaching 75% of the starting body weight. Both male and female mice were used at random for all control and infected treatment groups. All animal experiments were conducted in accordance with Duke IACUC.

### Isolation of respiratory epithelial cells for scRNA-seq

Six mice were infected with 75,000 PFU of Mal04-Cre, and two were administered PBS for mock controls. The lungs were collected for mock, 3, 14, and 28 dpi (one mouse of each sex for each condition). To isolate the murine respiratory epithelial cells for fluorescence-activated cell sorting (FACS), the lungs were perfused with PBS and inflated with dispase (Corning, catalog no. 354235 or StemCell, catalog no. 07913) and 1% low-melt NuSieve GTG agarose (Lonza; catalog no. 50081) dissolved in water. The lungs were then incubated in dispase for 45 min at room temperature before being minced in Dulbecco’s Modified Eagle Medium with DNase I (Sigma-Aldrich; catalog no. D4527-200KU). The cell suspension was homogenized further by vigorous pipetting and filtered using a 70 µm cell strainer (Olympus; catalog no. 25-376). A 1× Pharm Lyse buffer (BD Biosciences; catalog no. 555899) was used to lyse red blood cells from the suspension. PBS with 2% bovine serum albumin (BSA) (Lampire; catalog no. 7500855) was used to neutralize the lysis buffer. Next, the cells were stained for FACS. The following antibodies were used: CD45 (BioLegend; clone 30-F11; 1:100) and CD326/EpCAM (BD Biosciences; clone G8.8; 1:100). Cells were incubated with antibodies for 1 h on ice in the dark. The samples were then sorted by the Duke Cancer Institute using BDFACSDiVa. Approximately 15,000 EpCAM+/CD45−/tdTomato + cells (infected and survivor cells) and 15,000 EpCAM+/CD45−/tdTomato- cells (uninfected and bystander cells) were collected from each time point. The cell suspensions were counted using a Nexcelom Cellometer K2, and the cells were diluted to capture about 30,000 cells per sample. The two samples from the same time point were pooled as one sample, and then cells were submitted to the Duke University Molecular Genomics Core for 10× scRNA-seq. The samples were prepared by the core using Chromium Single-cell 3′ Reagent Kit. The raw and normalized reads are accessible at NCBI GEO with accession number GSE275873. The Illumina base call files were demultiplexed into FASTQs, then aligned to custom concatenated mm10 and Mal04-Cre genome and counted using 10× Genomics CellRanger pipeline (version 3.1.0). This created a new count matrix, which was then analyzed in R utilizing the R package Seurat.

### Data QC

The raw and filtered output files from CellRanger were read into SoupX (v1.6.2) to filter reads from lysed cells. Contaminated reads were estimated using the command autoEstCont. Those reads were used to make the adjusted count table using the command adjustCounts to make new matrices. The matrices for each individual sample were examined for initial QC. Cells that contained fewer than 1,000 or more than 5,000 genes were excluded from analysis. Cells with fewer than 2,000 and more than 65,000 unique molecular identifiers (UMIs) were also removed. Additionally, cells that had more than 10% mitochondrial genes, more than 12% ribosomal genes, or more than 8% immune genes were removed as contaminants from sorting. Additionally, sex-specific genes were removed from analysis, as well as any cells that did not express *Epcam*. After initial QC, all the samples were combined for initial unbiased clustering using the standard Seurat pipeline. Clusters of ciliated cells were identified using markers *Foxj1, Tuba1a*, and *Ccdc153*; secretory cells used markers *Scgb1a1, Chad*, and *Retnla*; AT1 pneumocytes used markers *Pdpn*, *Hopx*, and *Ager*; and AT2 pneumocytes used markers *Sftpc*, *Elovl1*, and *Chil1*.

### Infected sample analysis

The 3 dpi samples were combined into one Seurat object. Then, the object was further subsetted to make a new Seurat object of infected cells. Infected cells were defined as cells that expressed at least one influenza segment at a level higher than what was determined as background with the mock uninfected samples. All other cells from the 3 dpi samples were considered uninfected bystander cells. Genes identified by the FindMarkers function in Seurat were put into PANTHER (pantherdb.org) for the GO pathway analysis.

### Survivor and bystander sample analysis

The 14 dpi samples were combined into one Seurat object, and 28 dpi samples were combined into one separate Seurat object. Cells were survivor cells if they expressed tdTomato at a higher level than what was determined as background with the mock infected samples. All other cells are bystander cells. The objects were combined in various ways and unbiasedly clustered utilizing the Seurat workflow.

### AT2 sample analysis

AT2 pneumocytes from each of the time points were subsetted into new Seurat objects. AT2 pneumocytes were defined as cells that express *Sftpc* but do not express any *Scgb1a1*, *Hopx*, or *Foxj1*. The objects were combined in various ways and unbiasedly clustered utilizing the Seurat workflow. Genes identified by the FindMarkers function in Seurat were used for the GO pathway analysis.

### Confocal microscopy

Mouse lungs were perfused with PBS and inflated with 1:1 Tissue-Tek OCT (VWR; catalog no. 25608–930) and 8% PFA and tied off with suture string. The lungs were removed from the mouse and placed in 4% PFA at 4°C overnight for fixation. The lungs were then submerged in 30% sucrose in PBS at 4°C for at least 24 h before embedding in OCT. Once the lungs were in OCT, they were cryosectioned (10 µm sections). Sections were blocked with Blocking One Histo (Nacalai Tesque; catalog no. 06349–64) for 20 min at room temperature. The sections were then stained with primary antibodies overnight at 4°C and incubated with secondary antibodies at room temperature for 1 h (1:1000). Primary antibodies used include Pro-Sftpc (Millipore; catalog no. ab3786, 1:250) and ZO-1 (Thermo Fisher; catalog no. 33-9100, 1:500). Following incubation with a secondary antibody, lung sections were treated with the Vector TrueVIEW Autofluorescence Quenching Kit (VectorLabs; catalog no. SP-8400-15) for 30 min. Afterwards, the nuclei were stained using Hoechst 33342 (1:10,000) or 4′,6-diamidino-2-phenylindole (1:10,000). Following nuclei staining, sections were washed in PBS and mounted using Prolong Diamond Antifade Mountant (Invitrogen; catalog no. P36965). Images were captured utilizing a Zeiss upright confocal microscope, and raw images were processed using Fiji software (NIH).

### Isolation of AT2 pneumocytes

Mice were infected with 75,000 PFU of Mal04-Cre, and lungs were collected 14 dpi. Single-cell suspensions were generated, as described in STAR Protocols (34). The cell suspensions were next stained for fluorescence-activated cell sorting. The following antibodies were used: CD326/EpCAM (BioLegend; clone G8.8, 1:200), Lysotracker-Green DND-26 (Thermo Fisher Scientific; catalog no. L7526, 1:10000), CD31-eFluor450 (BioLegend; clone 390; 1:200), and CD45-eFluor450 (BioLegend; clone 30-F11, 1:200). Single EpCAM+/CD31−/CD45−/Lysotracker-Green+/tdTomato+ (survivor AT2) and EpCAM+/CD31-/CD45−/Lysotracker-Green+/tdTomato- cells (bystander AT2) cells were sorted using the Beckman Coulter MoFlo Astrios EQ High-speed Cell Sorter, the Sony MA900 Cell Sorter, or the Sony SH800 Cell Sorter.

### Culturing AT2 pneumocytes *ex vivo*

The collected cells were diluted to a concentration of 2,000 cells per 25 µL of media, as indicated in the published STAR protocol (34). An equal volume of Matrigel (Corning; catalog no. 354235) was added to the media, and the cell composition and 25–50 µL domes of cells embedded in Matrigel and media were plated on six-well plates. mIL1-B was added to the media for the first 4 days for P0 and the first 3 days for subsequent passages. It is not included in the media for the remainder of the period the alveolospheres are cultured. Images of alveolospheres were captured using Zeiss Axio Observer Z1 and analyzed and processed using Fiji software (NIH).

### Bulk RNA-seq sample preparation

Bystander and survivor AT2 pneumocytes were collected from three female mice and cultured *ex vivo*, as previously described. After 7 days, alveolospheres were dissociated, and total RNA was extracted from each sample utilizing the Monarch Total RNA Miniprep Kit (NEB, T2010S). RNA libraries were prepared utilizing NEBNext Ultra II RNA Library Prep Kit (E7770S) and NEBNext Multiplex Oligos for Illumina (E7335S). The samples were then submitted to the Sequencing and Genomics Technologies Core to be sequenced on an Illumina NovaSeq X Plus. The raw sequencing data are available at NCBI GEO under accession number GSE275874.

### Bulk RNA-seq analysis

Utilizing the CLC Genomics software, the raw reads were aligned to a custom concatenated mm10 and Mal04-Cre genome, and differential expression analysis between bystanders and survivors was performed. Genes with a max group mean above 1, log_2_fold change above 0.5, and a FDR *P*-value of less than 0.05 were used for further GO analysis in PANTHER.

### Statistical tests

The statistical tests used in each individual experimental analysis are noted in the figure legends and performed in GraphPad Prism (version 10.1.0).

## SUPPLEMENTAL MATERIAL

Supplemental caption mbio.02743-24-s0001.docx Caption for Table S1.

Table S1 mbio.02743-24-s0002.xlsx Gene ontology analysis performed.

ASM does not own the copyrights to Supplemental Material that may be linked to, or accessed through, an article. The authors have granted ASM a non-exclusive, world-wide license to publish the Supplemental Material files. Please contact the corresponding author directly for reuse.
