# Supplementary material for: Influenza B virus infection alters the regenerative potential of murine alveolar type 2 pneumocytes
Source: mBio. 2024 Dec 31;16(2):e02743-24. doi: 10.1128/mbio.02743-24 (PMC11796384; doi:10.1128/mbio.02743-24)
Supplement: Supplemental caption — Caption for Table S1. [file mbio.02743-24-s0001.docx]

**Supplemental Tables: Gene Ontology Analysis**

The supplemental tables show the complete gene ontology (GO) analysis results. The GO analysis corresponding to each figure can be seen labeled as individual tabs of the file. Tab 1: Fig2D.Upregulated_Bystanders. Tab 2: Fig2E.Upregulated_Infected. Tab 3: Fig3C.AT2-1_AT2-2. Tab4: Fig4J.Passaged_Survivors. The highlighted GO terms represent the terms that are displayed in each figure based on the parameters outlined in each specific figure legend.
